# Development and validation of a nomogram to predict postoperative delirium in older patients after major abdominal surgery: a retrospective case-control study

**DOI:** 10.1186/s13741-024-00399-3

**Published:** 2024-05-16

**Authors:** Yun-Gen Luo, Xiao-Dong Wu, Yu-Xiang Song, Xiao-Lin Wang, Kai Liu, Chun-Ting Shi, Zi-Lin Wang, Yu-Long Ma, Hao Li, Yan-Hong Liu, Wei-Dong Mi, Jing-Sheng Lou, Jiang-Bei Cao

**Affiliations:** 1 Department of Anesthesiology, the First Medical Center, Chinese PLA General Hospital, 28 Fuxing Road, Beijing, 100853 China; 2 Medical School of Chinese PLA, 28 Fuxing Road, Beijing, 100853 China; 3 Beidaihe Rest and Recuperation Center of People’s Liberation Army, Hebei, 066100 China; 4 National Clinical Research Center for Geriatric Diseases, Chinese PLA General Hospital, 28 Fuxing Road, Beijing, 100853 China

**Keywords:** Delirium, Nomogram, Older patients, Surgery, Risk factors

## Abstract

**Background:**

Postoperative delirium is a common complication in older patients, with poor long-term outcomes. This study aimed to investigate risk factors and develop a predictive model for postoperative delirium in older patients after major abdominal surgery.

**Methods:**

This study retrospectively recruited 7577 patients aged ≥ 65 years who underwent major abdominal surgery between January 2014 and December 2018 in a single hospital in Beijing, China. Patients were divided into a training cohort (*n* = 5303) and a validation cohort (*n* = 2224) for univariate and multivariate logistic regression analyses and to build a nomogram. Data were collected for 43 perioperative variables, including demographics, medical history, preoperative laboratory results, imaging, and anesthesia information.

**Results:**

Age, chronic obstructive pulmonary disease, white blood cell count, glucose, total protein, creatinine, emergency surgery, and anesthesia time were associated with postoperative delirium in multivariate analysis. We developed a nomogram based on the above 8 variables. The nomogram achieved areas under the curve of 0.731 and 0.735 for the training and validation cohorts, respectively. The discriminatory ability of the nomogram was further assessed by dividing the cases into three risk groups (low-risk, nomogram score < 175; medium-risk, nomogram score 175~199; high-risk, nomogram score > 199; *P* < 0.001). Decision curve analysis revealed that the nomogram provided a good net clinical benefit.

**Conclusions:**

We developed a nomogram that could predict postoperative delirium with high accuracy and stability in older patients after major abdominal surgery.

**Supplementary Information:**

The online version contains supplementary material available at 10.1186/s13741-024-00399-3.

## Background

Global aging is an increasing concern, and the percentage of older patients is expected to double over a 35-year period from 8.5% in 2015 (Janssen et al. 2019a, b). More than an estimated 12 million older patients need surgical procedures every year, and they are more susceptible than younger patients to postoperative complications (Karalapillai et al. 2020). Delirium, the most common postoperative complication in adults over the age of 65, is expected to increase simultaneously (Marcantonio 2017, Shi et al. 2019).

Postoperative delirium (POD) is an acute organic brain syndrome characterized by a transient disturbed attention span or altered levels of consciousness that can prolong the length of hospital stays and increase medical expenses (Abelha et al. 2013). Furthermore, patients with POD have been shown to have poorer long-term outcomes, including a reduction in cognition and quality of life, increased readmissions, and shorter survival (Abelha, Luís 2013, Aitken et al. 2017, Brown et al. 2016, Gleason et al. 2015). Therefore, it is necessary to identify risk factors for delirium and establish an effective risk-predicting model to prevent POD. A retrospective cohort study conducted by Ying et al. identified carbamazepine, hemoglobin, and blood urea nitrogen (BUN) as major risk factors for delirium (Wang et al. 2020). In addition, Bowman et al. identified 55 risk factors of delirium, including cognitive impairment or mental illness, psychoactive drugs, frailty, and infection (Bowman et al. 2020). However, these studies were conducted either in a single type of surgery with small sample sizes or in Western populations, which does not represent the growing aging population undergoing surgery in China.

In 2017, the European Society of Anesthesiology summarized the risk factors for POD, but these risk factors were not differentiated according to the type of surgery (Aldecoa et al. 2017). However, the occurrence of POD is known to be related to the type of surgery (Zipser et al. 2020), and major abdominal surgery, which is often performed on older patients, is associated with a high incidence of POD (Olin et al. 2005). Generally, major abdominal surgery is relatively complicated and is associated with a relatively large trauma burden, which can trigger a series of physiological stress reactions (Romain et al. 2020). The incidence of delirium after major abdominal surgery is reported to be 3–39% in the older population, which places a significant burden on society (Janssen et al. 2019a, b, Probst et al. 2017). Therefore, we conducted a retrospective study in older patients undergoing major abdominal surgery at the Chinese People’s Liberation Army (PLA) General Hospital to investigate the risk factors and construct a prediction model for POD.

## Methods

### Ethical review and waiver of consent

This study was conducted in accordance with the principles of the Declaration of Helsinki. Ethical approval for this study was provided by the Ethical Committee of the First Medical Center of the Chinese PLA General Hospital, Beijing, China (S2019-311-03). As this was a retrospective study of electronic medical records, the Medical Ethics Committee waived the need for informed consent. This manuscript adheres to the applicable STROBE guidelines.

### Study population

We selected patients who underwent major abdominal surgery at the Chinese PLA General Hospital between January 2014 and December 2018 using an electronic medical record system. The inclusion criteria were as follows: (1) age ≥ 65 years, (2) patients who underwent major abdominal surgery, and (3) surgical time was > 1 h and the patients were under general anesthesia.

The exclusion criteria were as follows: (1) patients diagnosed with preoperative delirium, consciousness disturbance, or psychiatric disorders, including epilepsy, anxiety, Alzheimer’s disease, Parkinson’s disease, schizophrenia, depression, hepatic encephalopathy, pulmonary encephalopathy, renal encephalopathy, or hydrocephalus; (2) patients with severe visual or hearing impairment preoperatively; (3) patients who underwent multi-site surgery; (4) patients died within 24 h postoperatively; and (5) patients with medical records missing > 50% of data.

### Surgery and anesthesia

Major abdominal surgery was defined as any type of surgery performed through abdominal access, expected to last >1 h, or with an expected blood loss > 500 mL involving hepatobiliary-pancreatic-gastrointestinal, urological, or gynecological systems. (Pensier et al. 2022, Shiao et al. 2009) During surgery, all patients underwent routine monitoring (including electrocardiogram, heart rate, pulse oximetry, and blood pressure) and general anesthesia. General anesthesia was maintained with sufentanil, remifentanil, propofol, sevoflurane, and rocuronium. Anesthetics were discontinued before the end of the operation, and sufentanil or non-steroidal analgesics, such as flurbiprofen axetil, were administered as needed.

### Diagnostic criteria for POD

POD was assessed using descriptions documented in medical records (Kuhn et al. 2014, Song et al. 2023). The inclusion criteria were as follows: (1) the postoperative medical records contained words such as “disorientation”, “poor sleep”, “confusion”, and other similar meaning words in Chinese; and (2) the medication prescription contained risperidone, olanzapine, droperidol, haloperidol, or quetiapine postoperatively. The exclusion criteria were as follows: the aforementioned symptoms and drugs were captured in the medical records preoperatively. The accuracy of the results was checked by two experienced neurologists who manually reviewed all medical records of patients with delirium based on the Diagnostic and Statistical Manual of Mental Disorders, Fifth Edition (DSM-IV) criteria.

### Data collection and variable selection

All patients, including patients undergoing emergency surgery, must complete common blood tests, blood biochemistry, liver and kidney function tests before surgery ensuring that all blood biochemical test results are obtained within 2 weeks before surgery. Data for analysis were extracted from electronic medical records including demographic, medical history, laboratory results, imaging data from the Picture Archiving and Communication System, and anesthetic data from the Anesthesia Information Management System.

### Statistical analysis

Continuous variables with normal distribution were presented as mean ± standard deviation and compared using Student’s t-test; alternatively, they were reported as medians (interquartile range [IQR]) and compared using the Mann–Whitney *U* test. Categorical data, reported as frequency (%), were compared using the chi-squared test or Fisher’s exact test. Independent risk factors for POD were then determined using univariate and multivariate logistic regression (LR) analyses in the training cohort. In the multivariate LR, each regression coefficient was converted proportionally on a 100-point scale. Next, we calculated the calibration curves, receiver operating characteristic (ROC) curves, and decision curve analysis of the nomogram for the two cohorts. Statistical significance was defined as *P* < 0.05. All tests were two-tailed. Data analysis was performed using R software (version 3.6.3; R Foundation, Vienna, Austria). Several packages were used within the R environment, including “mice,” “base,” “dplyr,” “Mass,” “pROC,” “ResourceSelection,” “rmda” and “Hmisc”.

## Results

### Characteristics of participants

**Fig. 1:**
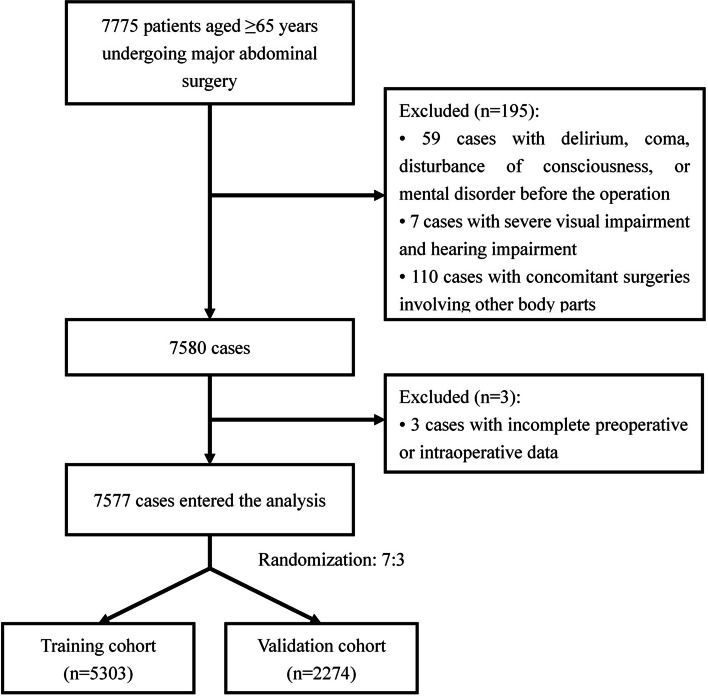
Flow diagram detailing the selection of patients included in the retrospective analysis

A total of 7577 patients were enrolled for analysis from January 2014 to December 2018, and 43 perioperative clinical variables were extracted from electronic medical records. There were 380 (5.02%) patients that met the diagnostic criteria of POD. Patients were randomly divided into training (*n* = 5303) and validation (*n* = 2274) cohorts at a ratio of 7:3 (Fig. 1). There were no significant differences in demographic or clinical characteristics between the two cohorts. Additional details are provided in Additional file 1. Additional file 2 shows the differences in clinical characteristics between patients with and without POD in the training cohort (*n* = 5303). Compared to patients without POD, patients with POD were older and had a lower body mass index (BMI). Patients who could not take care of themselves or had a history of chronic obstructive pulmonary disease (COPD) had a higher probability of developing POD. There were also differences in the surgical approaches. In a comparison of preoperative biochemical test results, patients with POD had lower hemoglobin, white blood cell count (WBC), glucose, total protein, and serum albumin levels, and higher BUN levels than those in the non-POD group. Analysis of surgery-related factors showed that patients with POD had a higher rate of emergency surgery and a higher American Society of Anesthesiologists (ASA) classification. Furthermore, patients who experienced POD had longer anesthesia and operative times, more blood loss and remifentanil consumption, a greater colloidal crystal ratio, a higher rate of blood transfusion, and a longer period of intraoperative hypotension (mean blood pressure ≤ 60 mmHg).

### Independent risk factors and the construction of a nomogram in the training cohort

**Fig. 2:**
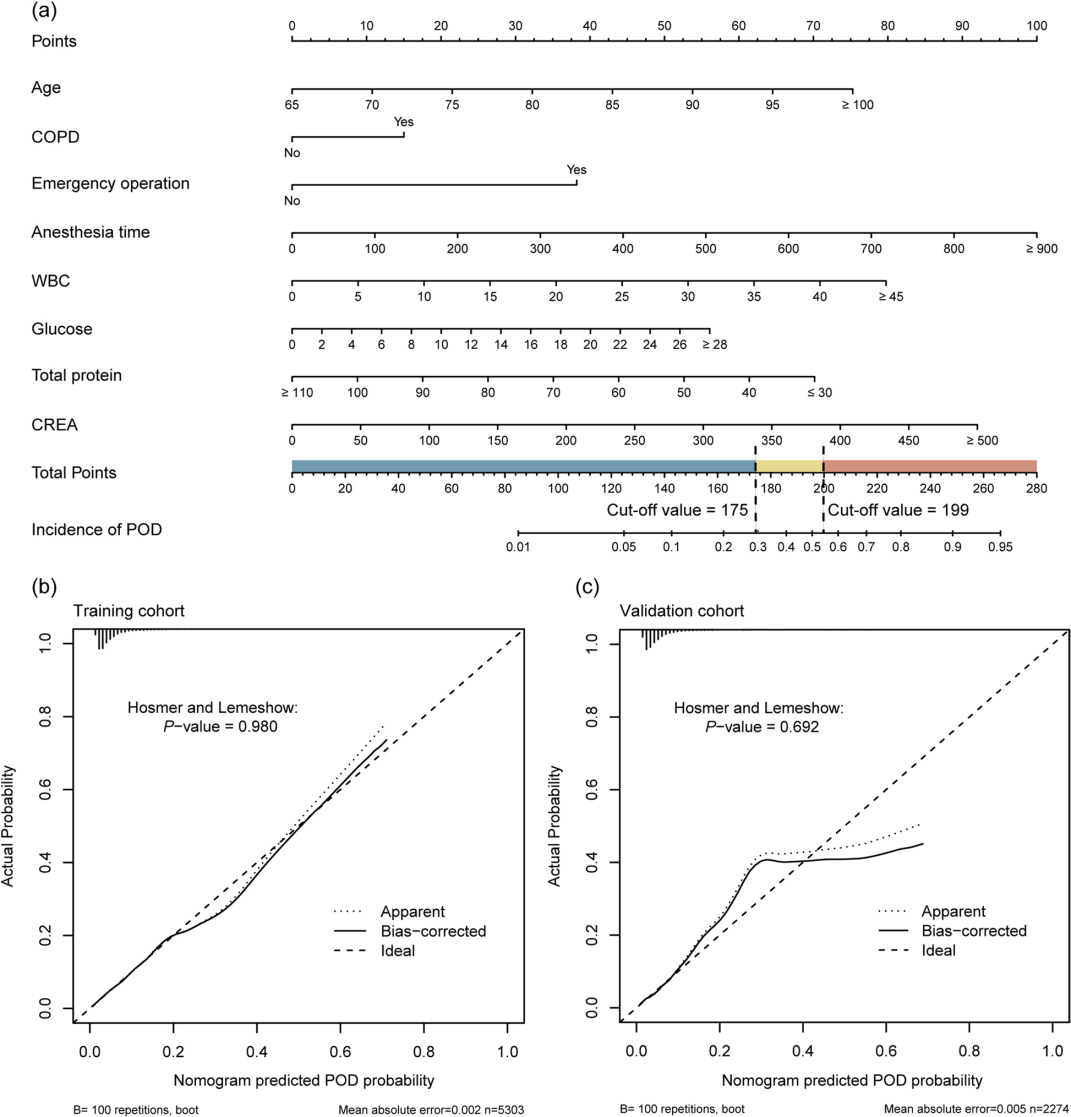
Nomogram for predicting postoperative delirium (POD) and calibration plots in the two cohorts. **a** nomogram, **b** calibration plot for training cohort, **c** calibration plot for validation cohort

Variables with significant differences between groups were included in the multivariate LR model. A stepwise LR method was used to screen variables with the smallest Akaike Information Criterion (AIC). Multivariate LR analysis revealed that age, COPD, WBC, glucose, total protein, creatinine, emergency surgery, and anesthesia time were independent risk factors for POD (Table 1). The complete results of univariate and multivariate analysis are presented in Additional file 3. Based on the scores for these independent risk factors, a nomogram was created to predict the incidence of POD (Fig. 2a).

**Table 1:** Univariate and multivariate analysis of independent risk factors for postoperative delirium in the training cohort

Variable	Univariate analysis			Multivariate analysis		
	*B*	OR (95%CI)	*p*	*B*	OR (95%CI)	*p*
Age	0.091	1.096 (1.072–1.119)	< 0.001	0.089	1.094 (1.069–1.119)	0.000
COPD	0.810	2.248 (1.426–3.403)	< 0.001	0.624	1.867 (1.140–2.933)	0.009
WBC	0.135	1.145 (1.101–1.19)	< 0.001	0.074	1.076 (1.030–1.125)	0.001
Glucose	0.119	1.126 (1.073–1.179)	< 0.001	0.083	1.087 (1.024–1.148)	0.004
Total protein	− 0.054	0.947 (0.929–0.966)	< 0.001	− 0.036	0.964 (0.945–0.983)	0.000
Creatinine	0.010	1.01 (1.006–1.015)	< 0.001	0.008	1.008 (1.003–1.012)	0.001
Emergency	2.147	8.561 (5.721–12.592)	< 0.001	1.59	4.901 (2.962–7.971)	0.000
Anesthesia time	0.003	1.003 (1.002–1.004)	< 0.001	0.005	1.005 (1.003–1.006)	0.000

*COPD* chronic obstructive pulmonary disease, *OR* odds ratio, *WBC* white blood cell count

### Performance of the nomogram

**Fig. 3:**
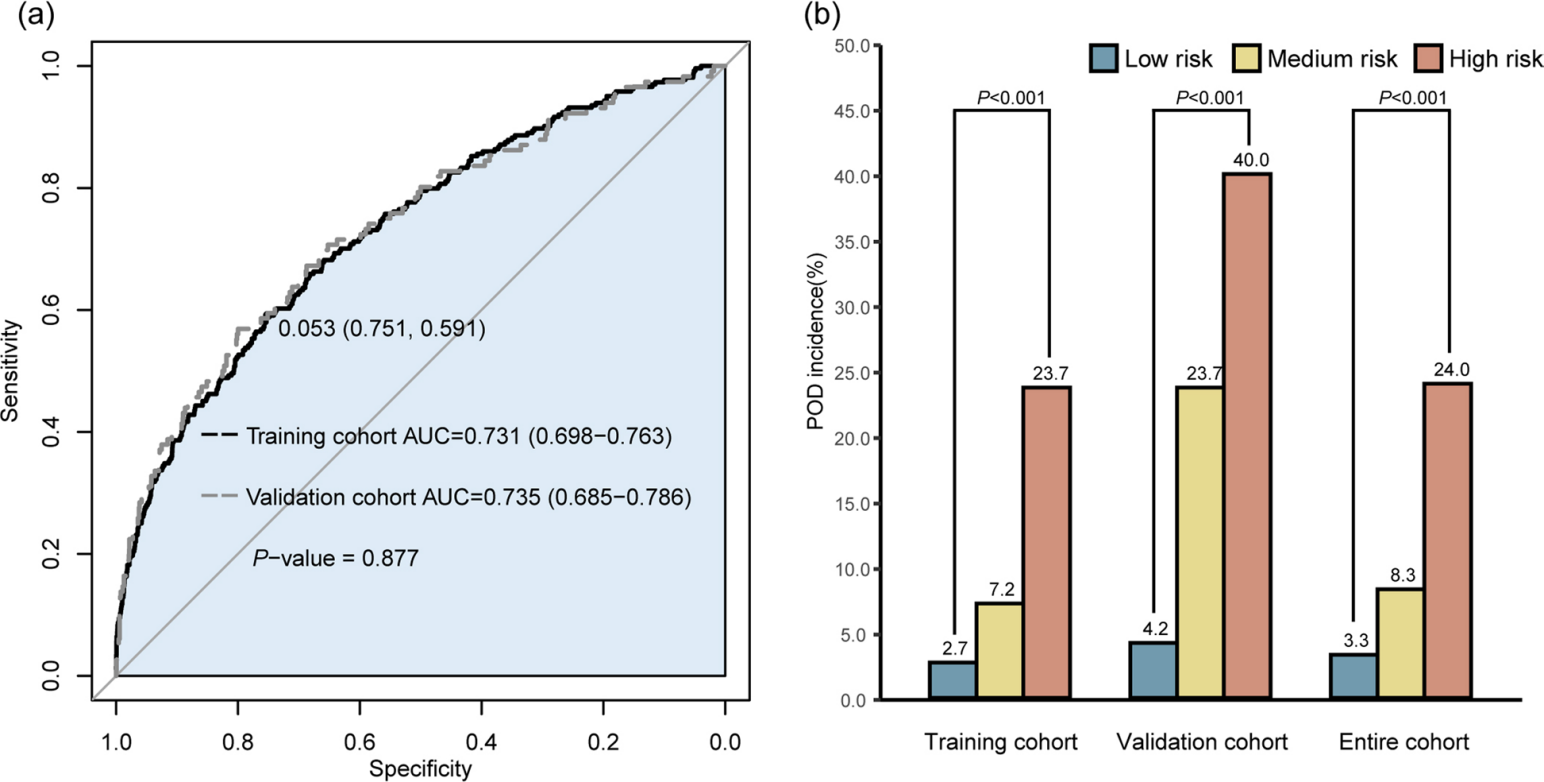
Performance of the nomogram for predicting POD. **a** ROC curves of the nomogram in training and validation cohorts, **b** The incidence of POD was compared between three groups in three cohorts

The calibration curves of the nomogram were in good agreement with the prediction of early POD in the training and validation cohorts (Fig. 2b, c). The *P* values of the Hosmer-Lemeshow goodness-of-fit test for the training and validation cohorts were 0.98 and 0.69, respectively. Based on the corresponding ROC curves, the model showed an area under the ROC curve (AUC) of 0.731 (95% confidence interval [CI] 0.698 to 0.763) and 0.735 (95% CI 0.685 to 0.786) (Fig. 3a). The optimal nomogram cut-off value for discriminating POD was 175.24. So patients with a total score of 175 points or less were categorized into the low-risk group. Then for patients with a total score above 175 points, we performed ROC curve analysis using the total score as the test variable and the actual frequency of POD as the status variable. The obtained cut-off value from the ROC curve was 198.96 points and was used to categorize them into medium-risk and high-risk groups. Additional Figure S1 shows the ROC curves of nomogram scores. The low-risk group had a nomogram score of < 175, and the medium-risk group had a nomogram score of 175~199 and the high-risk group had a score of > 199. The incidence of POD was significantly different between the three groups in all cohorts (*P* < 0.001, Fig. 3b).

### Practicability of the nomogram

**Fig. 4:**
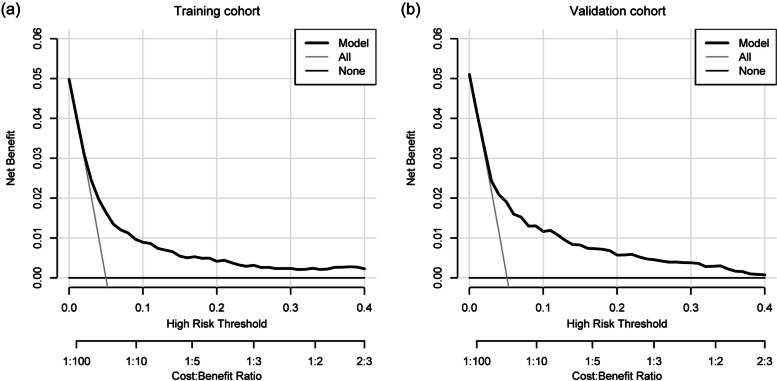
Decision curve analysis for the nomogram. **a** DCA of the nomogram in training cohort, **b** DCA of the nomogram in validation cohort

The decision curve analysis (DCA) demonstrated that the nomogram provided superior net benefits in the training and validation cohorts (Fig. 4). The nomogram was clinically useful in both cohorts. Collectively, these results indicate that the nomogram performed well in predicting the incidence of POD.

## Discussion

In this study, we developed and validated a nomogram for POD in older patients who underwent major abdominal surgery. The model included eight clinical variables that can be routinely obtained, namely age, COPD, WBC, glucose, total protein, creatinine, emergency surgery, and anesthesia time. The cut-off values were 175 and 199, and patients could be divided into low-risk, medium-risk, and high-risk groups based on the total nomogram score.

POD, the most common postoperative complication in older patients undergoing major abdominal surgery, has a complex pathophysiological mechanism and often indicates a poor prognosis (Hughes et al. 2020, Siddiqi et al. 2006). We identified age, COPD, emergency surgery, anesthesia time, WBC, glucose, total protein, and creatinine as independent risk factors for POD. According to previous studies, age is a definite risk factor for almost all types of surgeries (Hughes, Boncyk 2020). Yamamoto et al. revealed that emergency surgery was an independent predictor for sepsis-associated delirium (Yamamoto et al. 2020). However, previous studies have not definitively established an association between a history of COPD and POD. COPD is a systemic inflammatory disease; long-term hypoxemia and repeated infections have been shown to be significant factors in POD (Bowman, Jones 2020). In a prospective observational study, Cui et al. found that low cerebral tissue oxygen saturation (SctO_2_) may be associated with an increased risk of delirium (Cui et al. 2021). COPD may affect the occurrence of POD through this pathway. Additionally, extended anesthesia time implies greater levels of fluid input, more complicated surgery, more challenging intraoperative conditions, and the use of multiple drugs; and these factors are all associated with the occurrence of POD (Brown, LaFlam 2016).

We also found that four preoperative biochemical indicators (total protein, WBC, glucose, and creatinine) were closely associated with POD. Ishihara et al. found that serum albumin concentration (≤ 3.7 g/dL) was an independent risk factor for POD (Ishihara et al. 2021). Preoperative malnutrition is significantly associated with POD (Dong et al. 2023). Furthermore, globulin and WBC are mainly related to the immune function of the body and infection. Duceppe et al. found that active infection or sepsis, usually accompanied by elevated globulin and white blood cells, significantly increased the risk of delirium (Duceppe et al. 2019). Our current findings suggest that preoperative hyperglycemia is an independent risk factor for POD while preoperative diabetes is not; these findings are consistent with those by Windmann et al. who found that patients without diabetes with preoperative hyperglycemia may be at high risk for POD (Windmann et al. 2019). This indicates that perioperative blood glucose control is also vital for people without diabetes. In summary, these eight independent risk factors are relatively consistent with our clinical expectations, and the four perioperative biochemical indicators can provide potentially viable targets for further clinical intervention.

We developed and validated a predictive nomogram based on these eight predictors. Previous research has also attempted to establish predictive models of POD. Racine et al. failed to establish a prediction model for POD in older patients after non-cardiac surgery using various statistical methods (Racine et al. 2021). Wong et al. confirmed that hospital-acquired delirium can be predicted using machine learning methods (Wong et al. 2018); however, machine learning relies on a large number of clinical variables to improve the accuracy of prediction. More importantly, a model built using machine learning cannot guide targeted interventions, which greatly affects its clinical application. It is worth mentioning that Li et al. developed a prediction model for POD in a population across all age groups undergoing major abdominal surgery. The model contained six predictors, including age, history of arrhythmia, history of mental illness or cognitive impairments, albumin levels, coagulation function, and surgical Apgar score. The surgical Apgar score was calculated by intraoperative estimated blood loss, lowest mean arterial pressure, and lowest heart rate (Li et al. 2021). Their model involved nine variables, as the surgical Apgar score could not be obtained easily. Additionally, older patients represented only a low proportion (35.3%) of their training cohort. Compared with the model developed by Li et al., our model was developed with a much larger sample (*n* = 7577) involving the older population. Our model does not perform as well as their model in terms of AUC; however, our model is more applicable in clinical practice. First, POD mainly occurs in the older population, resulting in serious outcomes; therefore, we developed our model specifically for the older population. Second, in terms of variable selection, our model was more suitable for clinical application. The indicators included in our model (age, COPD history, emergency surgery, and anesthesia time) are relatively easy to access, while the other four biochemical parameters (WBC, glucose, total protein, and creatinine) are objectively obtained through routine preoperative blood testing, with only a small risk of error. Our findings imply that the identification of more effective biomarkers for delirium will lead to greater progress in the prediction of delirium.

The occurrence of POD prolongs the length of hospitalization for older patients and increases the average inpatient costs. It has been suggested that a fair amount of POD can be prevented (up to 40% in some studies) and multicomponent interventions are useful in POD prevention measures and postoperative recovery (Chen et al. 2017; Clemmesen et al. 2018; Hughes, Boncyk 2020; Humeidan et al. 2021, Ito et al. 2019, Khan et al. 2020, Perbet et al. 2018). The model we developed may facilitate the clinical application of these interventions and provide potential intervention indicators for POD treatment in the future. At the same time, our model may contribute to the postoperative rehabilitation of older patients and obtain better health and economic benefits.

Similar to other retrospective studies, this study may not accurately reflect the incidence of POD and preoperative patient status. The overall incidence of POD (~ 5%) for major abdominal surgery in older adults is low. This is likely the consequence of a retrospective approach used to diagnose POD. Hyperactive or mixed cases of POD were detected, whereas hypoactive cases of POD, which accounted for a large proportion, were missed with the employed retrospective approach. However, a series of medical interventions are applied for hyperactive or mixed cases of POD; therefore, the model we developed meets the requirement for clinical application. On the other hand, we did not use variables of perioperative pain, which are important risk factors for POD, since it would be difficult to obtain this information retrospectively. For the same reason, we were unable to validate the model described by Li et al. using our dataset because we excluded patients with a preoperative history of mental illness or cognitive impairment. Considering this, a multicenter prospective study will be useful to further validate the predictive value of our study. Therefore, we have initiated a multicenter, large-scale, prospective study on POD in older patients.

In conclusion, our study identified eight variables (age, COPD, emergency surgery, anesthesia time, WBC, glucose, total protein, and creatinine) as independent risk factors for POD in older patients after major abdominal surgery and developed a nomogram that was capable of predicting POD with high degrees of accuracy and stability.

### Supplementary Information

Additional file 1: Demographic and clinical characteristics of the training and validation cohorts (*N*=7577)

Additional file 2: Demographic and clinical characteristics of the training cohort according to the delirium status (*N*=5303)

Additional file 3: Univariate and multivariate analysis of risk factors for postopertaive delirium in the training cohort

Additional file 4: Additional Figure S1. ROC curves of nomogram scores (a. The cut-off value for low-risk and medium-risk groups was 175. b. The cut-off value for medium-risk and high-risk groups was 199)

## Data Availability

No datasets were generated or analysed during the current study.

## Abbreviations

AIC: Akaike Information Criterion
AUC: Area under curve
BMI: Body mass index
BUN: Blood urea nitrogen
COPD: Chronic obstructive pulmonary disease
LR: Logistic regression
POD: Postoperative delirium
PLA: People’s Liberation Army
ROC: Receiver operating characteristic
WBC: White blood cell count
