# Supplementary material for: Development and validation of a nomogram to predict postoperative delirium in older patients after major abdominal surgery: a retrospective case-control study
Source: Perioper Med (Lond). 2024 May 16;13:41. doi: 10.1186/s13741-024-00399-3 (PMC11100071; doi:10.1186/s13741-024-00399-3)
Supplement: Supplementary file 1 — Additional file 1: Demographic and clinical characteristics of the training and validation cohorts (N=7577) [file 13741_2024_399_MOESM1_ESM.docx]

| Additional file 1  No  Yes  P-value | | | |
| --- | --- | --- | --- |
| Demographic and clinical characteristics of the training and validation cohorts (N=7577) | | | |
| Variables | Training cohort  (N=5303) | Validation cohort  (N=2274) | *P*-value |
| Age(years) | 70(67~74) | 70(67~74) | 0.598 |
| BMI(kg/m^2^) | 23.46(21.34~25.65) | 23.565(21.48~25.65) | 0.652 |
| Gender, n(%) |  |  | 0.599 |
| Male | 3391(63.9%) | 1439(63.3%) |  |
| Female | 1912(36.1%) | 835(36.7%) |  |
| Comorbidities and/or past history | | | |
| Smoking, n(%) |  |  | 0.518 |
| No | 3802(71.7%) | 1613(70.9%) |  |
| Yes | 1501(28.3%) | 661(29.1%) |  |
| Alcohol use, n(%) |  |  | 0.451 |
| No | 3813(71.9%) | 1615(71.0%) |  |
| Yes | 1490(28.1%) | 659(29.0%) |  |
| Self-care, n(%) |  |  | 0.068 |
| No | 5216(98.4%) | 2222(97.7%) |  |
| Yes | 87(1.6%) | 52(2.3%) |  |
| Hypertension, n(%) |  |  | 0.975 |
| No | 3103(58.5%) | 1329(58.4%) |  |
| Yes | 2200(41.5%) | 945(41.6%) |  |
| Diabetes mellitus, n(%) |  |  | 0.709 |
| No | 3988(75.2%) | 1720(75.6%) |  |
| Yes | 1315(24.8%) | 554(24.4%) |  |
| Coronary heart disease, n(%) |  |  | 0.903 |
| No | 4873(91.9%) | 2087(91.8%) |  |
| Yes | 430(8.1%) | 187(8.2%) |  |
| Cerebrovascular disease, n(%) |  |  | 0.189 |
| No | 4759(89.7%) | 2017(88.7%) |  |
| Yes | 544(10.3%) | 257(11.3%) |  |
| Cirrhosis of the liver, n(%) |  |  | 0.529 |
| No | 4982(93.9%) | 2127(93.5%) |  |
| Yes | 321(6.1%) | 147(6.5%) |  |
| Malignant tumor, n(%) |  |  | 0.999 |
| No | 495(9.3%) | 213(9.4%) |  |
| Yes | 4808(90.7%) | 2061(90.6%) |  |
| COPD, n(%) |  |  | 0.996 |
| No | 5054(95.3%) | 2168(95.3%) |  |
| Yes | 249(4.7%) | 106(4.7%) |  |
| Premedication | | | |
| sleeping pills, n(%) |  |  | 0.43 |
| No | 4599(86.7%) | 1988(87.4%) |  |
| Yes | 704(13.3%) | 286(12.6%) |  |
| atropine, n(%) |  |  | 0.616 |
| No | 3240(61.1%) | 1404(61.7%) |  |
| Yes | 2063(38.9%) | 870(38.3%) |  |
| Preoperative test results | | | |
| HGB(g/L) | 126(112~138) | 126(112.25~138) | 0.959 |
| WBC(*10^9^/L) | 5.81(4.78~7.07) | 5.83(4.81~7.14) | 0.344 |
| Platelet(*10^9^/L) | 209(169~259) | 212(168~263) | 0.133 |
| Glucose(mmol/L) | 5.14(4.65~6.07) | 5.15(4.65~6.02) | 0.971 |
| Total protein(g/L) | 66(62.1~70) | 66(62.1~70.2) | 0.948 |
| Serum albumin(g/L) | 38.5(35.8~41) | 38.6(35.8~41.1) | 0.585 |
| BUN(mmol/L) | 5.02(4.08~6.23) | 4.96(4.08~6.1) | 0.447 |
| CREA(μmol/L) | 71(60.3~82.55) | 71.15(60~82.6) | 0.627 |
| Total bilirubin(μmol/L) | 11.5(8.3~17.7) | 11.3(8.3~17.575) | 0.432 |
| Direct bilirubin(μmol/L) | 3.5(2.4~6.1) | 3.5(2.3~6) | 0.352 |
| ALT(U/L) | 15.5(10.4~30.2) | 15.4(10.4~29.8) | 0.909 |
| Serum K^+^(mmol/L) | 4(3.78~4.26) | 4.01(3.78~4.28) | 0.207 |
| Surgery-related factors | | | |
| Emergency, n(%) |  |  | 0.941 |
| No | 5164(97.4%) | 2213(97.3%) |  |
| Yes | 139(2.6%) | 61(2.7%) |  |
| Surgical approach, n(%) |  |  | 0.944 |
| Open | 2590(48.8%) | 1116(49.1) |  |
| Laparoscopic | 1966(37.1%) | 834(36.7%) |  |
| Da Vinci Robot | 747(14.1%) | 324(14.2%) |  |
| Anesthesia time(min) | 235(187~300) | 237(185.25~297.75) | 0.785 |
| Surgical time(min) | 185(137~245) | 185(140~240) | 0.543 |
| ASA classification, n(%) |  |  | 0.288 |
| Ⅰ | 32(0.6%) | 23(1%) |  |
| Ⅱ | 4101(77.3%) | 1761(77.4%) |  |
| Ⅲ | 1106(20.9%) | 462(20.3%) |  |
| Ⅳ | 48(0.9%) | 18(0.8%) |  |
| Ⅴ | 16(0.3%) | 10(0.4%) |  |
| Urine output(ml) | 300(150~600) | 300(150~600) | 0.841 |
| Bleeding(ml) | 100(50~200) | 100(50~200) | 0.529 |
| Fluid volume(ml) | 2650(2100~3440) | 2600(2100~3300) | 0.349 |
| Colloidal crystal ratio | 0.312(0.222~0.455) | 0.312(0.217~0.455) | 0.44 |
| Blood transfusion, n(%) |  |  | 0.45 |
| No | 4503(84.9%) | 1947(85.6%) |  |
| Yes | 800(15.1%) | 327(14.4%) |  |
| Autologous blood, n(%) |  |  | 0.972 |
| No | 5273(99.4%) | 2262(99.5%) |  |
| Yes | 30(0.6%) | 12(0.5%) |  |
| Sufentanil dose(μg) | 50(40~60) | 50(40~60) | 0.639 |
| Remifentanil dose(mg) | 2.104(1.449~2.828) | 2.123(1.453~2.835) | 0.776 |
| Time of SBP$\geq$140mmHg  )) | 15(5~40) | 15(5~40) | 0.193 |
| Time of DBP$\geq$90mmHg | 0(0~5) | 0(0~5) | 0.77 |
| Time of MBP$\leq$60mmHg | 5(0~15) | 5(0~15) | 0.094 |
| Note: BMI, body-mass index; ICU, intensive care unit; HGB, hemoglobin; WBC, white blood cell count; BUN, blood urea nitrogen; CREA, creatinine. | | | |
