# Supplementary material for: Development and validation of a nomogram to predict postoperative delirium in older patients after major abdominal surgery: a retrospective case-control study
Source: Perioper Med (Lond). 2024 May 16;13:41. doi: 10.1186/s13741-024-00399-3 (PMC11100071; doi:10.1186/s13741-024-00399-3)
Supplement: Supplementary file 2 — Additional file 2: Demographic and clinical characteristics of the training cohort according to the delirium status (N=5303) [file 13741_2024_399_MOESM2_ESM.docx]

| Additional file 2  No  Yes  P-value | | | |
| --- | --- | --- | --- |
| Demographic and clinical characteristics of the training cohort across delirium status (N=5303) | | | |
| Variables | Delirium group (N=264) | Non-delirium group (N=5039) | *P*-value |
| Age(years) | 73(68~78) | 70(67~74) | <.001 |
| BMI(kg/m^2^) | 22.86(20.8~25.7) | 23.51(21.4~25.7) | 0.081 |
| Gender, n(%) |  |  | 0.312 |
| Male | 177(67.0%) | 3214(63.8%) |  |
| Female | 87(33.0%) | 1825(36.2%) |  |
| Comorbidities and/or past history | | | |
| Smoking, n(%) |  |  | 0.503 |
| No | 184(69.7%) | 3618(71.8%) |  |
| Yes | 80(30.3%) | 1421(28.2%) |  |
| Alcohol use, n(%) |  |  | 0.348 |
| No | 197(74.6%) | 3616(71.8%) |  |
| Yes | 67(25.4%) | 1423(28.2%) |  |
| Self-care, n(%) |  |  | <.001 |
| No | 250(94.7%) | 4966(98.6%) |  |
| Yes | 14(5.3%) | 73(1.4%) |  |
| Hypertension, n(%) |  |  | 0.307 |
| No | 146(55.3%) | 2957(58.7%) |  |
| Yes | 118(44.7%) | 2082(41.3%) |  |
| Diabetes mellitus, n(%) |  |  | 0.657 |
| No | 195(73.9%) | 3793(75.3%) |  |
| Yes | 69(26.1%) | 1246(24.7%) |  |
| Coronary heart disease, n(%) |  |  | 0.628 |
| No | 240(90.9%) | 4633(91.9%) |  |
| Yes | 24(9.1%) | 406(8.1%) |  |
| Cerebrovascular disease, n(%) |  |  | 0.08 |
| No | 228(86.4%) | 4531(89.9%) |  |
| Yes | 36(13.6%) | 508(10.1%) |  |
| Cirrhosis of the liver, n(%) |  |  | 0.357 |
| No | 252(95.5%) | 4730(93.9%) |  |
| Yes | 12(4.5%) | 309(6.1%) |  |
| Malignant tumor, n(%) |  |  | 0.687 |
| No | 27(10.2%) | 468(9.3%) |  |
| Yes | 237(89.8%) | 4571(90.7%) |  |
| COPD, n(%) |  |  | <.001 |
| No | 239(90.5%) | 4815(95.6%) |  |
| Yes | 25(9.5%) | 224(4.4%) |  |
| Premedication | | | |
| sleeping pills, n(%) |  |  | 0.007 |
| No | 214(81.1%) | 4385(87.0%) |  |
| Yes | 50(18.9%) | 654(13.0%) |  |
| atropine, n(%) |  |  | 0.979 |
| No | 162(61.4%) | 3078(61.1%) |  |
| Yes | 102(38.6%) | 1961(38.9%) |  |
| Preoperative test results | | | |
| HGB(g/L) | 119.5(103.75~133) | 126(113~138) | <.001 |
| WBC(*10^9^/L) | 6.33(5.15~8.195) | 5.78(4.77~7.01) | <.001 |
| Platelet(*10^9^/L) | 211(170.75~269) | 208(169~259) | 0.55 |
| Glucose(mmol/L) | 5.415(4.738~7.17) | 5.12(4.64~6.03) | <.001 |
| Total protein(g/L) | 64.9(59.8~69.35) | 66.1(62.2~70.1) | <.001 |
| Serum albumin(g/L) | 37.15(33.68~40.13) | 38.6(35.9~41.1) | <.001 |
| BUN(mmol/L) | 5.41(4.318~7.01) | 5.01(4.07~6.2) | <.001 |
| CREA(μmol/L) | 73.7(61.775~92.85) | 70.9(60.2~82.2) | 0.001 |
| Total bilirubin(μmol/L) | 11.35(7.8~16.825) | 11.5(8.3~17.8) | 0.48 |
| Direct bilirubin(μmol/L) | 3.65(2.5~6.8) | 3.5(2.4~6) | 0.192 |
| ALT(U/L) | 14.85(9.2~26.525) | 15.5(10.5~30.4) | 0.044 |
| Serum K^+^(mmol/L) | 4.07(3.84~4.325) | 3.99(3.77~4.25) | 0.006 |
| Surgery-related factors | | | |
| Emergency, n(%) |  |  | <.001 |
| No | 225(85.2%) | 4939(98.0%) |  |
| Yes | 39(14.8%) | 100(2.0%) |  |
| Surgical approach, n(%) |  |  | <.001 |
| Open | 162(61.4%) | 2428(48.2%) |  |
| Laparoscopic | 72(27.3%) | 1894(37.6%) |  |
| Da Vinci Robot | 30(11.4%) | 717(14.2%) |  |
| Anesthesia time(min) | 259.5(201~335.75) | 235(185~297) | <.001 |
| Surgical time(min) | 205(154.75~280) | 185(135~245) | <.001 |
| ASA classification, n(%) |  |  | <.001 |
| Ⅰ | 1(0.4%) | 31(0.6%) |  |
| Ⅱ | 160(60.6%) | 3941(78.2%) |  |
| Ⅲ | 87(33.0%) | 1019(20.2%) |  |
| Ⅳ | 10(3.8%) | 38(0.8%) |  |
| Ⅴ | 6(2.3%) | 10(0.2%) |  |
| Urine output(ml) | 400(150~712.5) | 300(150~600) | 0.019 |
| Bleeding(ml) | 150(100~300) | 100(50~200) | <.001 |
| Fluid volume(ml) | 3000(2405~3600) | 2600(2100~3410) | 0.009 |
| Colloidal crystal ratio | 0.35(0.24~0.49) | 0.312(0.22~0.46) | <.001 |
| Blood transfusion, n(%) |  |  | <.001 |
| No | 186(70.5%) | 4317(85.7%) |  |
| Yes | 78(29.5%) | 722(14.3%) |  |
| Autologous blood, n(%) |  |  | 0.001 |
| No | 258(97.7%) | 5015(99.5%) |  |
| Yes | 6(2.3%) | 24(0.5%) |  |
| Sufentanil dose(μg) | 50(30,60) | 50(40,60) | 0.188 |
| Remifentanil dose(mg) | 2.305(1.474~3.143) | 2.1(1.448~2.808) | 0.015 |
| Time of SBP$\geq$140mmHg  )) | 20(5~45) | 15(5~40) | 0.043 |
| Time of DBP$\geq$90mmHg | 0(0~5) | 0(0~5) | 0.933 |
| Time of MBP$\leq$60mmHg | 10(0~25) | 5(0~15) | 0.004 |
| Note: BMI, body-mass index; ICU, intensive care unit; HGB, hemoglobin; WBC, white blood cell count; BUN, blood urea nitrogen; CREA, creatinine. | | | |
