# Supplementary material for: Development and validation of a nomogram to predict postoperative delirium in older patients after major abdominal surgery: a retrospective case-control study
Source: Perioper Med (Lond). 2024 May 16;13:41. doi: 10.1186/s13741-024-00399-3 (PMC11100071; doi:10.1186/s13741-024-00399-3)
Supplement: Supplementary file 3 — Additional file 3: Univariate and multivariate analysis of risk factors for postopertaive delirium in the training cohort [file 13741_2024_399_MOESM3_ESM.docx]

| Additional file 3 | | | | | | |
| --- | --- | --- | --- | --- | --- | --- |
| Univariate and multivariate analysis of risk factors for POD in the training cohort | | | | | | |
|  | Univariate analysis | | | Multivariate analysis | | |
| Variable | B | OR [95%CI] | *p* | B | OR [95%CI] | *p* |
| Age, per years | 0.091 | 1.096(1.072-1.119) | <0.001 | 0.089 | 1.094[1.069,1.119] | 0.000 |
| BMI, per kg/m^2^ | -0.034 | 0.967(0.931-1.003) | 0.076 |  |  |  |
| Gender, male vs. female | -0.144 | 0.866(0.663-1.122) | 0.282 |  |  |  |
| Comorbidities and/or past history | | | | | | |
| Smoking, yes vs. no | 0.102 | 1.107(0.841-1.444) | 0.46 |  |  |  |
| Alcohol use, yes vs. no | -0.146 | 0.864(0.646-1.142) | 0.314 |  |  |  |
| Self-care, yes vs. no | 1.338 | 3.81(2.038-6.635) | <0.001 |  |  |  |
| Hypertension, yes vs. no | 0.138 | 1.148(0.894-1.471) | 0.278 |  |  |  |
| Diabetes mellitus, yes vs. no | 0.074 | 1.077(0.808-1.42) | 0.605 |  |  |  |
| Coronary heart disease, yes vs. no | 0.132 | 1.141(0.723-1.721) | 0.549 |  |  |  |
| Cerebrovascular disease, yes vs. no | 0.342 | 1.408(0.965-1.999) | 0.065 |  |  |  |
| Cirrhosis of the liver, yes vs. no | -0.316 | 0.729(0.382-1.259) | 0.294 |  |  |  |
| Malignant tumor, yes vs. no | -0.107 | 0.899(0.608-1.383) | 0.609 |  |  |  |
| COPD, yes vs. no | 0.810 | 2.248(1.426-3.403) | <0.001 | 0.624 | 1.867[1.140,2.933] | 0.009 |
| Premedication | | | | | | |
| sleeping pills, yes vs. no | 0.449 | 1.567(1.128-2.136) | 0.006 |  |  |  |
| atropine, yes vs. no | -0.012 | 0.988(0.765-1.272) | 0.927 |  |  |  |
| Preoperative test results | | | | | | |
| HGB, per g/L | -0.016 | 0.984(0.978-0.99) | <0.001 |  |  |  |
| WBC, per *10^9^/L | 0.135 | 1.145(1.101-1.19) | <0.001 | 0.074 | 1.076[1.030,1.125] | 0.001 |
| Platelet, per *10^9^/L | 0.000 | 1(0.999-1.002) | 0.562 |  |  |  |
| Glucose, per mmol/L | 0.119 | 1.126(1.073-1.179) | <0.001 | 0.083 | 1.087[1.024,1.148] | 0.004 |
| Total protein, per g/L | -0.054 | 0.947(0.929-0.966) | <0.001 | -0.036 | 0.964[0.945,0.983] | 0.000 |
| Serum albumin, per g/L | -0.109 | 0.897(0.872-0.923) | <0.001 |  |  |  |
| BUN, per mmol/L | 0.108 | 1.114(1.064-1.166) | <0.001 |  |  |  |
| CREA, per μmol/L | 0.010 | 1.01(1.006-1.015) | <0.001 | 0.008 | 1.008[1.003,1.012] | 0.001 |
| Total bilirubin, per μmol/L | 0.001 | 1.001(0.998-1.002) | 0.548 |  |  |  |
| Direct bilirubin, per μmol/L | 0.001 | 1.001(0.998-1.003) | 0.526 |  |  |  |
| ALT, per U/L | -0.001 | 0.999(0.996-1.001) | 0.337 |  |  |  |
| Serum K^+^, per mmol/L | 0.486 | 1.625(1.174-2.235) | 0.003 |  |  |  |
| Surgery-related factors | | | | | | |
| Emergency, yes vs. no | 2.147 | 8.561(5.721-12.592) | <0.001 | 1.59 | 4.901[2.962,7.971] | 0.000 |
| Surgical approach |  |  |  |  |  |  |
| Open |  | Ref |  |  |  |  |
| Laparoscopic | -0.563 | 0.57(0.427-0.754) | <0.001 |  |  |  |
| Da Vinci Robot | -0.467 | 0.627(0.413-0.92) | 0.022 |  |  |  |
| Anesthesia time, per min | 0.003 | 1.003(1.002-1.004) | <0.001 | 0.005 | 1.005[1.003,1.006] | 0.000 |
| Surgical time, per min | 0.003 | 1.003(1.002-1.004) | <0.001 |  |  |  |
| ASA classification |  |  |  |  |  |  |
| Ⅰ |  | Ref |  |  |  |  |
| Ⅱ | 0.23 | 1.259(0.268-22.474) | 0.821 |  |  |  |
| Ⅲ | 0.973 | 2.647(0.558-47.393) | 0.341 |  |  |  |
| Ⅳ | 2.099 | 8.158(1.444-153.836) | 0.051 |  |  |  |
| Ⅴ | 2.923 | 18.6(2.741-374.445) | 0.01 |  |  |  |
| Urine output, per ml | 0.001 | 1.001(1-1.001) | <0.001 |  |  |  |
| Bleeding, per ml | 0.000 | 1(1-1.001) | <0.001 |  |  |  |
| Fluid volume, per ml | 0.000 | 1(1-1) | <0.001 |  |  |  |
| Colloidal crystal ratio | 1.267 | 3.552(2.233-5.612) | <0.001 |  |  |  |
| Blood transfusion, yes vs. no | 0.919 | 2.507(1.894-3.291) | <0.001 |  |  |  |
| Autologous blood, yes vs. no | 1.581 | 4.859(1.788-11.254) | <0.001 |  |  |  |
| Sufentanil dose, per μg | -0.007 | 0.993(0.988-0.999) | 0.019 |  |  |  |
| Remifentanil dose, per mg | 0.175 | 1.191(1.073-1.319) | <0.001 |  |  |  |
| Time of SBP$\geq$140mmHg, per min | 0.004 | 1.004(1.001-1.007) | 0.016 |  |  |  |
| Time of DBP$\geq$90mmHg, per min | 0.004 | 1.004(0.994-1.013) | 0.393 |  |  |  |
| Time of MBP$\leq$60mmHg, per min | 0.012 | 1.012(1.007-1.016) | <0.001 |  |  |  |
| Note: BMI, body-mass index; ICU, intensive care unit; HGB, hemoglobin; WBC, white blood cell count; BUN, blood urea nitrogen; CREA, creatinine. | | | | | | |
