# Supplementary figures and images for: Development and validation of a nomogram to predict postoperative delirium in older patients after major abdominal surgery: a retrospective case-control study
Source: Perioper Med (Lond). 2024 May 16;13:41. doi: 10.1186/s13741-024-00399-3 (PMC11100071; doi:10.1186/s13741-024-00399-3)

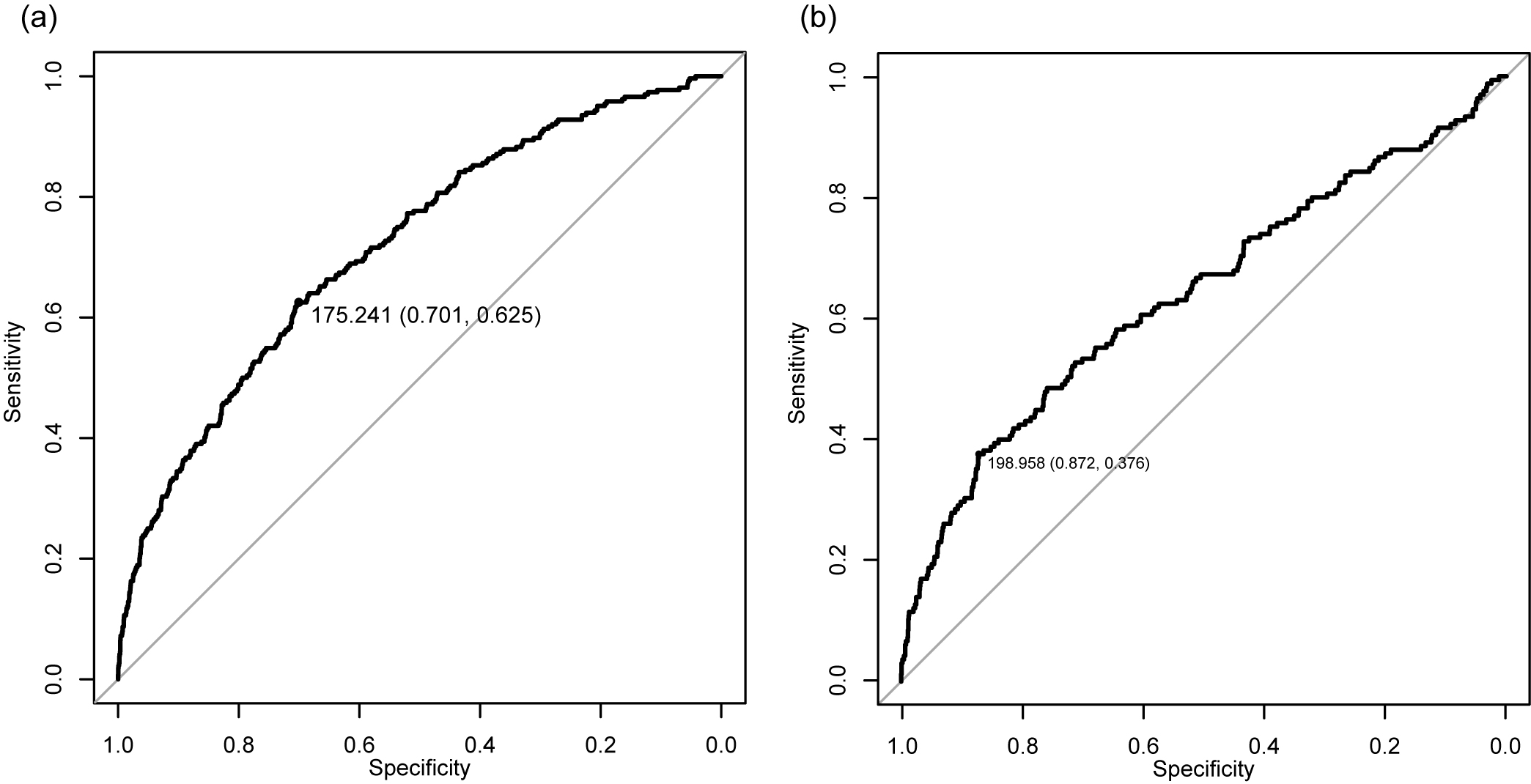

Supplement: Supplementary file 4 — Additional file 4: Additional Figure S1. ROC curves of nomogram scores (a. The cut-off value for low-risk and medium-risk groups was 175. b. The cut-off value for medium-risk and high-risk groups was 199) [file 13741_2024_399_MOESM4_ESM.jpg]
